# A scalable new model of germline cancer genomic care delivery: Assessing psychological outcomes

**DOI:** 10.1016/j.gim.2026.102537

**Published:** 2026-02-12

**Authors:** Ilana B. Solomon, Patrick Boyd, Yi Xiao, Kathryn R. Dang, Ashley Mochizuki, Madeline Currey, Michael Garcia, Marilan Luong, Frances Cheung, Stephen B. Gruber, Heather Hampel, Sarah Labib, Angela Mak, Ran Qiao, Sarah Tang, Sandra Davey, Stacy W. Gray

**Affiliations:** 1 Division of Clinical Cancer Genomics, City of Hope National Medical Center, Duarte, CA; 2 Department of Medical Oncology and Therapeutics Research, City of Hope National Medical Center, Duarte, CA; 3 Center for Precision Medicine, City of Hope National Medical Center, Duarte, CA; 4 Beckman Research Institute, Duarte, CA; 5 University of California, Los Angeles, David Geffen School of Medicine, Los Angeles, CA; 6 Transplantation and Cellular Therapy, Children’s Hospital Los Angeles, Los Angeles, CA; 7 University of Arizona College of Medicine, Phoenix, AZ; 8 Virginia Tech Carilion School of Medicine, Roanoke, VA; 9 California Northstate University, College of Medicine, Elk Grove, CA; 10 System Strategy Office, City of Hope National Medical Center, Duarte, CA

**Keywords:** Genetic services, Hereditary cancer syndromes, Oncology, Psychological outcomes

## Abstract

**Purpose:**

New care models promise to increase access to germline genetic testing. To decrease testing gaps, we offered universal germline testing without pretest genetic counseling to unselected patients at a comprehensive cancer center. To address safety concerns over the elimination of pretest counseling, we examined detrimental psychological impact (DPI) after participants underwent testing in this novel model.

**Methods:**

We assessed participants’ genomic testing-specific DPI using the Feelings About Genomic Testing Results instrument (2-weeks/6-months after disclosure). Overall DPI, negativity, uncertainty, positivity, and privacy concerns were assessed. We used chi-square, analysis of covariance multivariable regression, and mediation analysis to examine relationships between variables.

**Results:**

A total of 846 participants completed the survey. Most participants (96.0%) exhibited low DPI, negativity (95.6%), uncertainty (94.0%), and concerns about privacy (95.6%) and high positivity (55.6%). Participants with cancer had higher negativity and uncertainty than those without. DPI significantly diminished over time in participants with positive tests.

**Conclusion:**

Overall DPI was low, demonstrating that most participants are at minimal psychological risk when receiving germline results without pre-test counseling. However, a small subset of participants exhibit high distress and uncertainty. Additional efforts are needed to identify at-risk participants receiving universal testing who would benefit from interventions aimed at mitigating DPI.

## Introduction

Germline genetic testing enables personalized cancer risk assessment for patients and their family members. Subsequent risk-reduction and prevention strategies for individuals at increased genetic risk have been shown to improve patient outcomes.^1–3^ Additionally, genetic testing is increasingly utilized to guide targeted therapy in oncology.^4^

Given that most individuals with inherited cancer risk have not been diagnosed, many experts and professional groups support testing more broadly.^5^ Current testing standards include a reliance on guidelines to determine which individuals meet criteria based on personal and family history. However, this approach has proven to be insufficient. Studies show that approximately half of the patients that harbor actionable variants are missed by guidelines.^2,6^ Broad testing in cancer is increasingly becoming essential given that many cancer patients, who do not meet clinical criteria for testing, harbor germline findings that can direct cancer therapy. Shortcomings in guideline-based testing have moved the field toward universal testing approaches and new models of care delivery.^7,8^

As demand for genetic services increases, the traditional model of pre- and posttest genetic counseling becomes infeasible because of limited access to genetics clinics, a shortage of genetic counselors, and logistical constraints (eg, time required to refer/schedule).^9–11^ Recently, a large randomized clinical trial explored omitting pretest counseling and showed that levels of distress were noninferior in the arm without pretest counseling.^12^ Moreover, there were no significant differences in anxiety, depression, or decisional regret.

To increase access to germline genetic testing, we began offering germline testing to all patients through a broadly implemented Institutional Review Board-approved protocol, without formal pretest counseling in 2020. We administered a survey to a subset of participants to assess any detrimental psychological impact (DPI) of test result disclosure. We hypothesized that a very high proportion (eg, ≥90%) receiving germline genomic sequencing results would exhibit low DPI and negative emotions. Second, we hypothesized that although most participants (eg, ≥90%) would experience low uncertainty, the proportion of participants with high uncertainty would be higher than those exhibiting high DPI or negative emotions. Third, we hypothesized that participants with cancer (affected) would exhibit relatively higher DPI compared with those who have no history of cancer (unaffected) and that participants with pathogenic/likely pathogenic (PLP) germline results would exhibit higher overall DPI relative to those receiving negative results and variants of uncertain significance (VUS). We defined unaffected as anyone without a history of cancer based on the electronic medical record. As an exploratory aim, we assessed the relationship between type of result, DPI, and reported sharing of germline genomic test results with a family member.

## Materials and Methods

Our sampling frame included participants from the City of Hope Implementing Next-Generation Sequencing for Precision Intervention and Risk Evaluation (INSPIRE) Study. The INSPIRE study enrolls eligible COH patients regardless of personal and/or family history of cancer. Study consent is conducted in person or remotely by research staff. We elicit consent for institutional biobanking of leftover biospecimens, data use, and permission for future contact. The study informed consent form includes a brief overview of germline genetic testing for cancer and noncancer conditions, potential implications of testing for family members, and protections related to genetic discrimination. Participants can opt-in for germline testing for hereditary cancer (150+ panel) and an actionable disorders panel (American College of Medical Genetics and Genomics [ACMG] 59 v.2).^13^ All genetic test results are reviewed by a licensed genetic counselor. Actionable results (PLP in cancer risk genes and ACMG 59 genes) are disclosed by phone and a posttest counseling session is offered. If patients are not able to be reached after 2 phone attempts, results are returned by letter including a recommendation to schedule posttest counseling. Nonactionable results (ie, negative, VUS, and Heterozygotes for Autosomal Recessive conditions) are communicated via letter or electronic health record message with the option of genetic counseling.

### Study population and design

Patients were eligible for the study if they were enrolled in INSPIRE, had results in Epic Genomics, spoke English, and had a valid email. Participants were excluded if they received pretest genetic counseling before enrollment. We administered baseline (2 weeks) and follow-up (6 months) surveys after results disclosure through REDCap.^14,15^ We did not provide an incentive for participation. To ensure participant safety, a genetic counselor or genetic counseling assistant followed up by phone for all participants that (1) scored high in negativity (*n* = 37) and/or (2) requested follow-up in the comments section of an open-ended question on the survey, and/or (3) shared confusion/uncertainty regarding their genetic testing results in the open-ended question on the survey.

### Measures

The primary outcome measure was overall genomic testing-specific DPI using a modified (see Supplemental Material 1) version of the Feelings About Genomic Testing Results (FACToR) instrument.^16^ Participants responded to 12 items using a 0–4 (not at all to a great deal) scale. Within the overall scale, there was a 3-item negativity subscale (eg, “How upset did you feel about your genetic test result?”; α =.72), a 3-item uncertainty subscale (eg, “How uncertain did you feel about what your genetic test result means for you?”; α =.73), a 4-item positive feelings subscale (eg, “How relieved did you feel about your genetic test result?”; α =.77), and a 2-item privacy subscale (eg, “How concerned did you feel that your genetic test result would affect your employment status?”; α =.75). After reverse scoring items within the positivity subscale, scores for the overall scale were summed to measure overall DPI which had acceptable reliability (α =.76), similar to each subscale. To assess sharing test results with a family member, we asked participants, “Since receiving your genomic test results, have you shared the information with any blood relatives?” All response options other than “Yes” were counted as not sharing. Any participants indicating they did not have any blood relatives were excluded from analyses examining that variable (*n* = 25).

### Statistical analyses

We used chi-square analyses to compare demographic characteristics between affected and unaffected participants. Testing variables included gender, ethnicity, race, age, and result type. Frequencies for DPI used the midpoint of the response scales for overall DPI (>24), negativity (>6), uncertainty (>6), privacy concerns (>4), and positivity (>8) to indicate high levels of each respective scale. Scores at or below the cutoffs were considered low. We also conducted chi-square analyses to determine if overall DPI and subscale scores differed by cancer status. We conducted multivariate regressions to determine predictors of overall DPI and FACToR subscales. We transformed result type, age, and race into binary variables for clarity of interpretation for multivariate analyses. We conducted between-subjects analyses of covariance to determine if germline genomic testing result type (PLP vs VUS vs Neg) and cancer status (affected vs unaffected) were associated with DPI after controlling for age, gender, ethnicity, and race. The first and focal analysis of our study was conducted using baseline responses, and the second used follow-up survey responses. We also conducted pairwise comparisons between result type within each cancer status group at baseline to probe significant main effects. We conducted a repeated measures between-subjects analysis to assess changes in DPI over time, using the same between-subjects variables and covariates. Finally, we performed a mediation analysis to determine if the relationship between having a PLP and reported sharing of test results with a family member was explained, in part, by DPI. To examine if DPI partially mediated the relationship between having a PLP vs not and reported sharing of germline genomic testing results with family members, we used PLP status as the predictor variable, DPI as the mediator, and sharing of genomic test results with a family member as the outcome. Control variables included age, gender, race ethnicity, cancer status, satisfaction with care received, and understanding of provider explanation of results. All analyses were conducted within SPSS and the PROCESS add-on for mediation.^17^

## Results

We collected survey data between July 2022 and October 2023. Of the 3385 participants invited to take the baseline survey, a total of 846 participants completed it (see Figure 1). Our response rate was 34% after removing invalid email addresses, participants that did not recall receiving genomic test results, and participants who did not complete at least one of the FACToR questions. Most participants were over 65 years old, had a history of cancer, female, White, and did not identify as Hispanic or Latino. For breakdown of germline results and other participant characteristics see Table 1.

### Overall DPI and FACToR subscale descriptives

Supporting our main hypothesis, the majority of participants exhibited low DPI (96%) and had low negativity, uncertainty, and concerns about privacy (all ≥ 94.0%). In line with our second hypothesis, high uncertainty frequencies were slightly greater than high negativity frequencies. For more specifics about FACToR scores see Figure 2, and for subscale breakdowns by participant characteristics (eg, result type and cancer status), see Supplemental Material 2.

### Multivariable regression analysis for FACToR scores

We found relatively higher DPI among participants with PLPs, those with a history of cancer, those younger than 65 years of age, and those who were Hispanic or Latino. Additionally, we found relatively higher negativity, uncertainty, and concerns about privacy for those with PLPs, in those younger than 65, and those who were Hispanic or Latino. Finally, we found that those who had a PLP, had a personal history of cancer, and were male, had lower overall positivity about their genetic testing results. For additional information, see Table 2.

### Overall DPI at 2-week baseline

At 2 weeks after germline genomics test result disclosure, overall DPI differed among affected vs unaffected participants and differed by result type. Specifically, participants with cancer had relatively higher DPI than those unaffected. Within both affected and unaffected groups, those with a PLP had relatively higher DPI than those with a VUS or negative result (see Figure 3). For additional result statistics, see Supplemental Material 2.

### DPI change scores

There was a significant effect of cancer status (*P* =.048) and result type (*P* =.010) on DPI across time points from 2-weeks to the 6-month follow-up. Participants affected with cancer had relatively higher overall DPI than those unaffected across both time points, and those with PLPs exhibited relatively higher DPI than those with a VUS or negative result. Importantly, we found that participants with cancer who had a PLP exhibited decreased DPI over time. Conversely, participants with cancer who had a VUS exhibited increased DPI over time (see Figure 4). For additional comparison statistics across time, see Supplemental Material 2.

### Relationship between PLP status and sharing results with family

We found those with a PLP were more likely to have higher overall DPI, and as expected, those with PLP variants were more likely to share their genomic test results with family members. Critically however, examining the impact of having a PLP on sharing of test results through DPI, having higher DPI was associated with decreased family sharing for individuals with PLPs. For additional mediation analyses statistics and Supplemental Figure 1, see Supplemental Material 2.

## Discussion

To scale delivery of germline genomic testing, our institution implemented a model of care delivery which removed formal pretest genetic counseling. Aligned with our primary hypothesis, most participants in our study had low DPI, negativity, and uncertainty after test result disclosure. These findings support the safety of delivering results through this model. Our results are not only consistent with smaller studies analyzing DPI of individuals undergoing hereditary cancer testing without formal pretest genetic counseling,^18,19^ but are also supported by a recent large, randomized trial on women with breast cancer, which showed noninferiority of arms without pretest genetic counseling.^12^ Our unselected population is unique in the inclusion of participants with and without any type of cancer at a comprehensive cancer center in Southern California.

Studies have consistently shown that genetic test result disclosure does not cause depression or anxiety in most individuals in cancer and noncancer settings, including in the context of disclosure of findings from genome sequencing and exome sequencing.^20,21^ Our results are consistent with the findings by Culver et al^22^ from a multiethnic cohort of participants who underwent multigene panel testing for hereditary cancer showing that a majority of participants rarely or never experienced negative emotions related to testing. However, in line with our second hypothesis, despite participants infrequently reporting high overall DPI (ie, above scale midpoint), negative emotions, and uncertainty, high uncertainty was slightly more common than high negativity and overall DPI.

Consistent with other studies, a small subset of individuals experienced high DPI in the form of distress and uncertainty.^23^ Further efforts should be made to identify which individuals are most at risk of having high DPI in the wake of receiving germline genomic test results. Based on our findings, we see relatively higher DPI in individuals who have PLPs, are younger, are Hispanic or Latino, and have cancer. Age has previously been found to be negatively associated with cancer-related distress and genetic testing-related distress after genetic testing,^24,25^ and Hispanic/Latino individuals have been found to generally have more concerns about genetic testing.^26^ Although the ability to speak English was an inclusion criteria for the current study, many Hispanic/Latino participants in our sample may have been bilingual. Multilingual individuals may be more susceptible to DPI if they have lower levels of genetic test result comprehension due to language barriers. Finally, there could have been other unmeasured variables contributing to DPI and related to Hispanic/Latino status that were not assessed in this study.

It has been previously reported that higher pretest distress, anxiety, and cancer worry are predictors of distress after germline testing for hereditary cancer.^27,28^ A possible future direction includes adding an assessment of distress and cancer worry before universal germline genetic testing. However, such an assessment may decrease the feasibility and uptake of universal testing. Although most participants had low DPI, we observed expected differences in DPI based on cancer status and the type of results they received. We found that affected participants reported higher overall DPI relative to those who were unaffected, supporting our third hypothesis. This finding begs the question of whether this group of affected individuals was aware of potential treatment or clinical trial opportunities that may come from the identification of a germline PLP. Additionally, for both participants with and without cancer, those who received PLPs also reported relatively higher DPI relative to those who had VUS and negative results. These results are consistent with other studies showing that individuals with PLPs have higher Multidimensional Impact of Cancer Risk Assessment (MICRA) scores including high distress and uncertainty.^24,29–32^

Examining DPI over time, we found that among cancer participants with a PLP status, DPI decreased, which is consistent with prior research indicating that distress among individuals with PLPs dissipates over time.^33–35^ Interestingly, we found that affected individuals with VUS results had increased DPI over time; however, the magnitude of these effects, although in the opposite direction of PLPs, was much smaller. As such, it may be important to further examine the impact of VUS results over time for participants who may delay thinking about or processing their germline results. This is particularly important because many newly diagnosed participants may be initiating treatment concurrently with germline testing; thus, they may not have enough cognitive and emotional bandwidth to tend to germline results that are uncertain. Overall rates of formal posttest counseling in our study were 62% for participants with PLP, 8% with VUS, and 11% with negative findings. These results also suggest patients with VUS results may benefit from proactive outreach overtime from providers as a “second-touch” counseling to assess their psychological state. Alternatively, these findings warrant considering the value of returning all VUS, particularly those off phenotype, given the potential risk for harm with no clinical benefit because they are not actionable.

When assessing participants’ willingness to share results with family members, we found that participants with PLP findings were more likely to share germline test results with family members, as expected. However, higher levels of DPI were associated with a decreased likelihood of sharing results with family member(s). Such results suggest that, although there is an incentive to share PLP test results with family members for a variety of reasons, those with high DPI after genetic testing may be less likely to communicate critical health information to their at-risk family members. As such, interventions aimed at mitigating high DPI for those at risk (eg, having a history of cancer, being younger, etc), should likely be implemented early in the testing process. These results corroborate a recent review that also found the emotional impact of learning genetic test results can be a barrier to sharing test results with family members.^36^

Our results highlight how increased access to and delivery of universal germline genomic testing can be performed safely in participants with and without a history of cancer, across and multiple cancer types. Knowing that a subset of participants may be at risk of having high levels of DPI, additional resources and support throughout germline testing processes should be offered to those at greatest risk to mitigate DPI and increase sharing of test information. Our findings also indicate that follow-up could focus on interventions and support for those with PLP results, a history of cancer, those who are younger, and/or Hispanic or Latino because they are more likely to have relatively higher DPI. At this time, it is unclear whether these characteristics warrant system-wide interventions, but further exploration is warranted. Of note, we would recommend prioritizing interventions for those with a PLP and cancer over age and ethnicity because their level of impact on DPI (Table 2).

Our study has several limitations. Although we invited consecutive participants to participate, over the study period, we did not conduct a random sample of all participants enrolled in the INSPIRE study. Furthermore, our response rate was relatively low (34%), limiting the generalizability of our results. Our lower response rate is likely attributed to the fact that the study was designed for scalability; we did not provide participants with incentives, and we relied on a “cold” email invitation, rather than direct staff contact, for recruitment. Furthermore, after examining demographic differences between respondents and nonrespondents due to nonrespondent inclusion in the larger INSPIRE study, we found that the distribution of demographic characteristics differed by race, age, result type, and ethnicity. However, the actual differences were minimal except for ethnicity, whereby being non-Hispanic/Latino was associated with a higher likelihood of being a respondent. As such, it is important to consider nonresponse bias in the context of our study results and specifically how being Hispanic/Latino, and other cultural contexts, may be associated with both a lower likelihood of responding to our survey, as well as higher likelihood of being associated with greater DPI. Additionally, we were unable to examine differences in DPI for genes of different penetrance due to our sample size. We also did not measure health literacy of the participants, which may have influenced understanding of the results and therefore affected DPI. Finally, although we tried to measure test-specific distress by using the FACTOR, it is always possible that the DPI captured more general distress, particularly among cancer participants, compared with test-specific distress.

Our study contributes to a growing body of literature that demonstrates the safety of providing genomic testing for patients, at scale, without formalized pretest counseling. For the few (4%) of individuals experiencing relatively higher levels of DPI, interventions may be needed to mitigate DPI in at-risk populations. Although DPI in and of itself merits attention, our results also indicate that participants with high DPI may be less likely to share test results with family members. As such, support and interventions aimed at mitigating DPI might subsequently increase cascade testing.

## Supplementary Material

All supplemental material

Additional Information

The online version of this article (https://doi.org/10.1016/j.gim.2026.102537) contains supplemental material, which is available to authorized users.

## Figures and Tables

**Figure 1:**
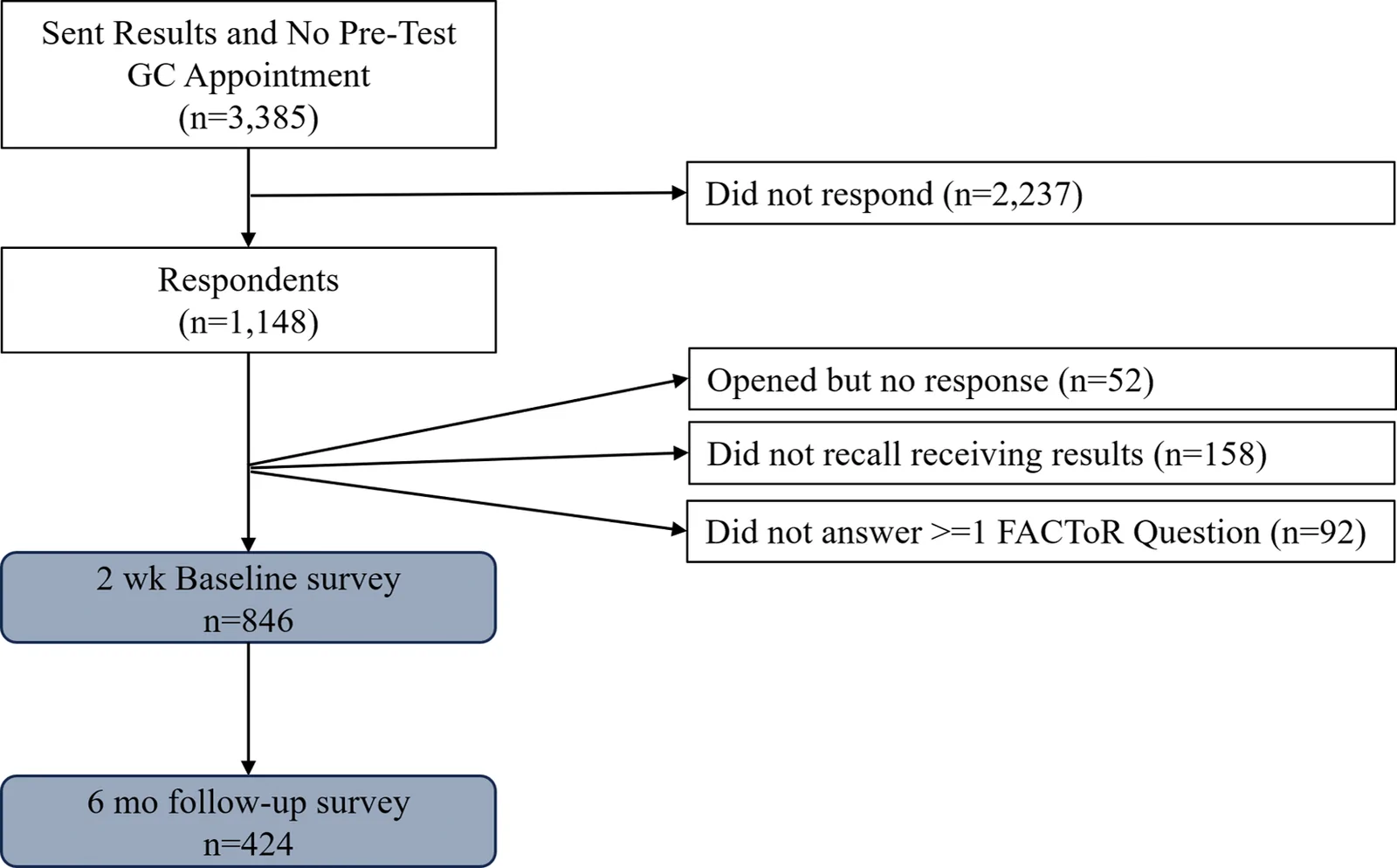
Survey study flow and participant progression.

**Figure 2:**
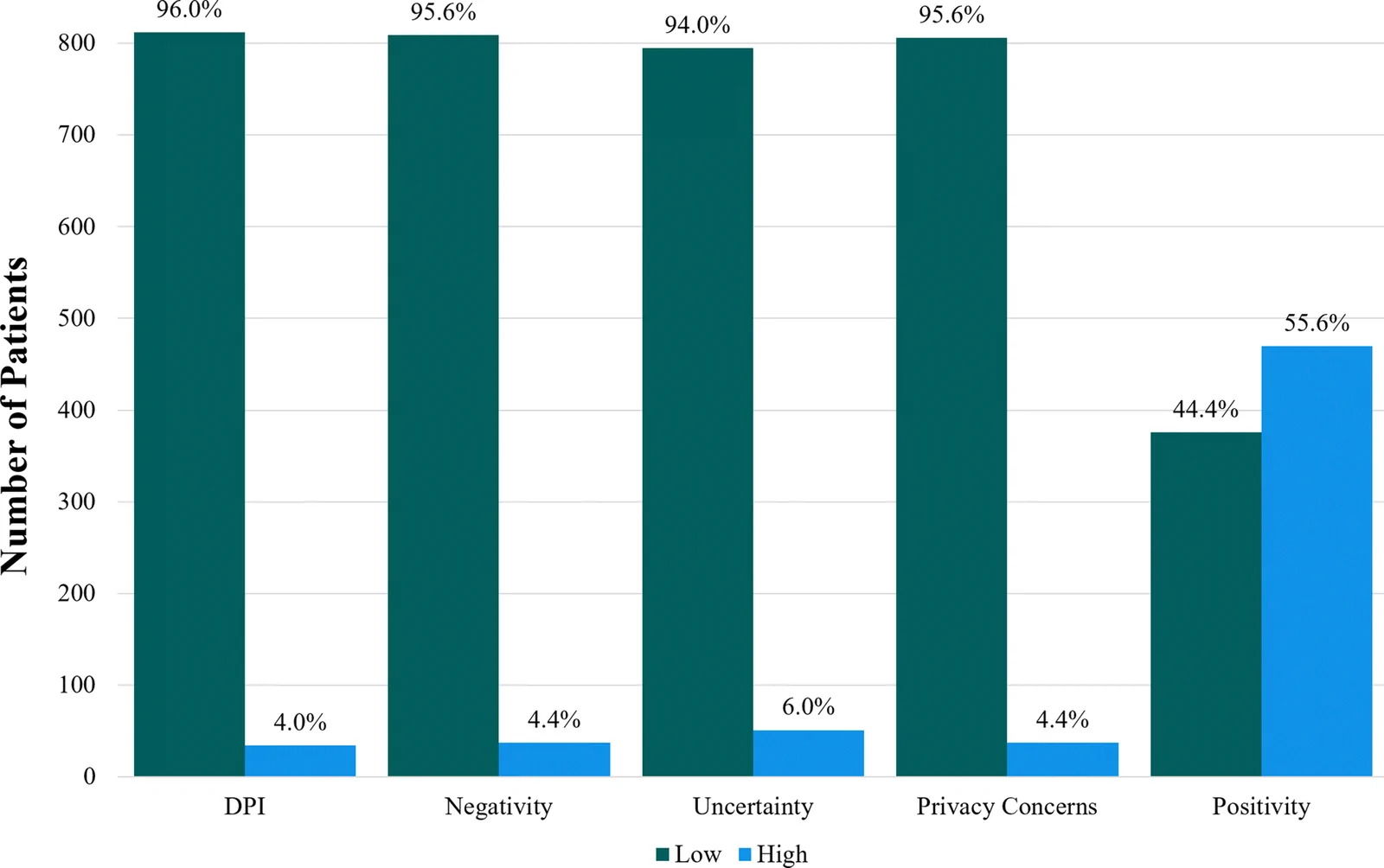
Frequencies of detrimental psychological impact and FACToR subscales. Note: Percentages are out of 846 total patients for each scale. Frequencies for DPI used the midpoint of the response scales for overall DPI (>24), negativity (>6), uncertainty (>6), privacy concerns (>4), and positivity (>8) to indicate high levels of each respective scale.

**Figure 3:**
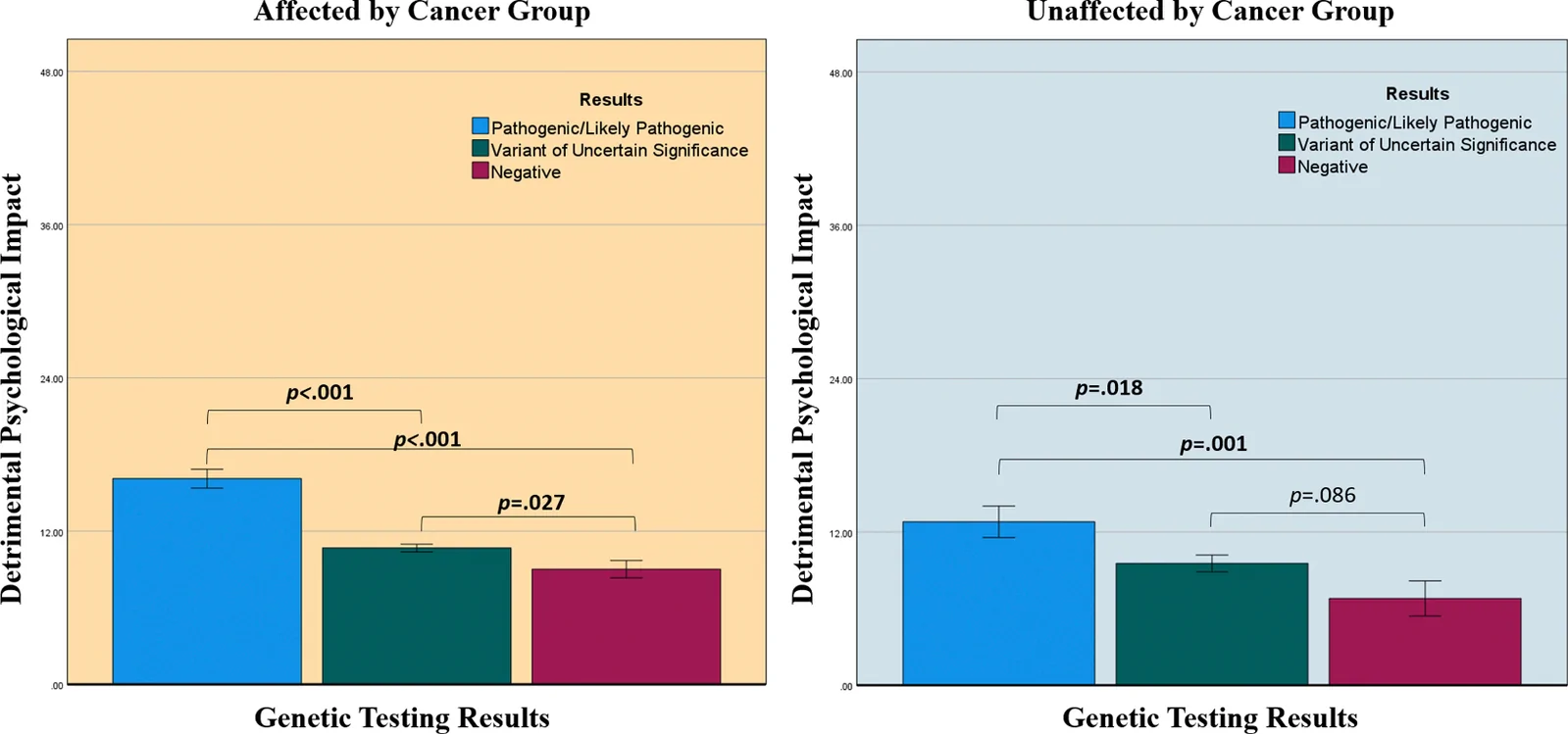
Detrimental psychological impact by cancer status and result type controlling for gender, ethnicity, race, and age. Note: Higher scores indicate more DPI. Mosaics and Heterozygote for AR conditions excluded. Bolded *P* values indicate significant differences between result type within cancer status groups. Additionally, DPI differed by result type *F*(2, 751) = 20.90, *P* <.001, η^2^ =.05, and cancer status *F*(1, 751) = 11.33, *P* <.001, η^2^ =.02.

**Figure 4:**
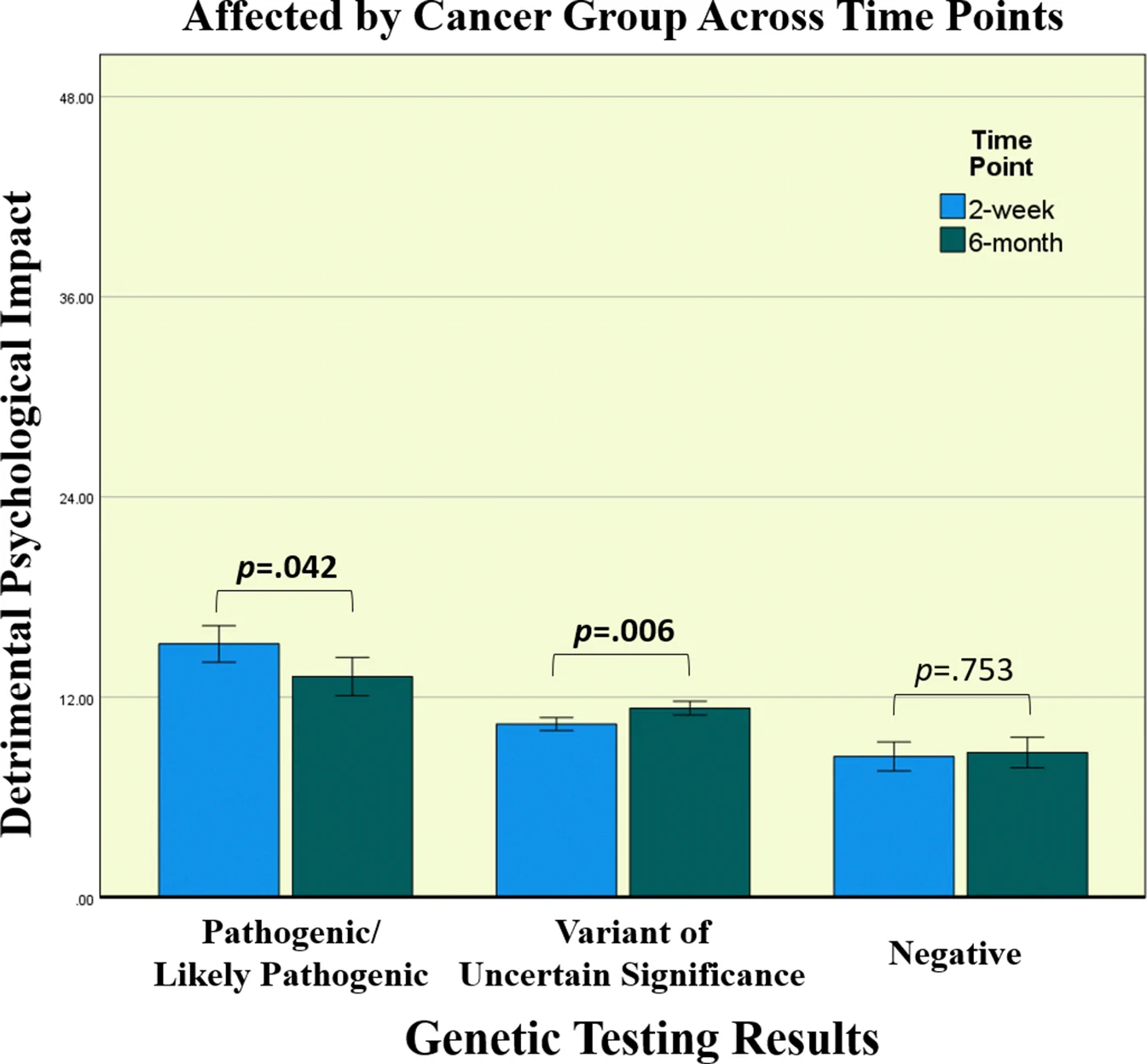
Detrimental psychological impact change scores for affected patients controlling for gender, ethnicity, race, and age.

**Table 1:** Demographics characteristics and differences between cancer status

Characteristics	Overall, *N* = 846	Affected, *N* = 690	Unaffected, *N* = 156	*P* Value^a^

Gender				.006
Female	572 (67.6%)	452 (65.5%)	120 (76.9%)	
Male	274 (32.4%)	238 (34.5%)	36 (23.1%)	
Ethnicity				.059
Not Hispanic or Latino	679 (80.3%)	559 (81.0%)	120 (76.9%)	
Hispanic or Latino	135 (16.0%)	110 (15.9%)	25 (16.0%)	
Unknown/Decline to answer	32 (3.8%)	21 (3.0%)	11 (7.1%)	
Race				.4
White	649 (76.7%)	523 (75.8%)	126 (80.8%)	
Asian	118 (13.9%)	103 (14.9%)	15 (9.6%)	
Black or African American	28 (3.3%)	25 (3.6%)	3 (1.9%)	
Other Race	11 (1.3%)	8 (1.2%)	3 (1.9%)	
American Indian or Alaska Native	6 (0.7%)	5 (0.7%)	1 (0.6%)	
Native Hawaii or Other Pac Islander	2 (0.2%)	2 (0.3%)	0 (0%)	
Unknown/Decline to answer	32 (3.8%)	24 (3.5%)	8 (5.1%)	
Age				<.001
18–39	24 (2.8%)	11 (1.6%)	13 (8.3%)	
40–64	344 (40.7%)	265 (38.5%)	79 (50.6%)	
65+	473 (56.4%)	413 (59.9)	64 (41.0%)	
Unknown/Decline to answer	1 (0.1%)	1 (0.1%)	-	
Clinical Significance				.2
VUS	548 (64.8%)	457 (66.2%)	91 (58.3%)	
Negative	112 (13.2%)	91 (13.2%)	21 (13.5%)	
PLP	102 (12.1%)	76 (11.0%)	26 (16.7%)	
Heterozygotes for AR conditions^b^	80 (9.5%)	63 (9.1%)	17 (10.9%)	
Mosaic^b^	4 (0.5%)	3 (0.4%)	1 (0.6%)	

a Pearson’s Chi-squared test; Fisher’s exact test; Wilcoxon rank sum test.

b Heterozygotes for AR conditions and Mosaics were excluded from any analysis comparing DPI by result type because of power concerns and lack of clinical actionability.

**Table 2:** FACToR parameter estimates at baseline for overall detrimental psychological impact and subscales

	Overall Detrimental Psych Impact	Positive Subscale	Negative Subscale	Uncertainty Subscale	Privacy Subscale

Variable	b, Est (95% CI)	b, Est (95% CI)	b, Est (95% CI)	b, Est (95% CI)	b, Est (95% CI)
Result Type: PLP vs non-PLP (ref)	**4.**96^d^ (3.57, 6.36)	−1.31^c^ (−0.43, −2.18)	2.02^d^ (1.58, 2.45)	1.19^d^ (1.69, 0.68)	0.33^b^ (0.66, 0.00)
Cancer: affected vs unaffected (ref)	1.64^c^ (0.46, 2.83)	−0.99^c^ (−1.73, −0.25)	0.07 (−0.30, 0.44)	0.38^a^ (−0.05, 0.81)	0.17 (−0.11, 0.45)
Age: <65 vs 65+ (ref)	1.57^d^ (0.61, 2.52)	−0.21 (−0.39, 0.81)	0.57^d^ (0.27, 0.86)	0.53^c^ (0.18, 0.88)	0.58^d^ (0.35, 0.80)
Sex: male vs female (ref)	0.78 (−0.22, 1.22)	−0.75^b^ (−1.38, −0.13)	−0.08 (−0.39, 0.23)	−0.07 (−0.43, 0.29)	−0.01 (−0.24, 0.23)
Race: non-white vs white (ref)	0.54 (−0.54, 1.62)	−0.10 (−0.77, 0.58)	0.10 (−0.24, 0.44)	0.14 (−0.26, 0.53)	0.20 (−0.06, 0.45)
Ethnicity: Hispanic vs nonHispanic (ref)	0.92^b^ (0.01, 1.83)	0.26 (−0.32, 0.83)	0.38^c^ (0.09, 0.66)	0.31^a^ (−0.02, 0.64)	0.45^d^ (0.24, 0.67)

Note: Reference groups (Result Type: 0 = VUS+Neg+Heterozygotes for AR conditions+Mosaics, 1 = PLP; Cancer Status: 0 = Unaffected, 1 = Affected; Age: 0 = 65+, 1 = younger than 65; Sex: 0 = female, 1 = male; Race: 0 = white, 1 = all other races; Ethnicity: 0 = not Hispanic or Latino, 1 = Hispanic or Latino). Mosaics and Heterozygotes for AR conditions were included in these analyses. Overall DPI range: 0–48. Positive subscale range: 0–16, Negative (distress) subscale range: 0–12, Uncertainty subscale range: 0–12, Privacy Concerns subscale range: 0–8. Positive subscale was reverse scored for inclusion in overall DPI scale but not as subscale. Higher scores on positive subscale indicate less PI, whereas higher scores for overall psych impact, negativity, uncertainty, and privacy concern subscales indicate greater impact.

a = *P* <.10.

b = *P* <.05.

c *P* <.01.

d = *P* <.001.

## Data Availability

Data are available upon individual request.
