# Supplementary material for: A scalable new model of germline cancer genomic care delivery: Assessing psychological outcomes
Source: Genet Med. Author manuscript; Available in PMC 2026 Oct 5. (PMC13638032; doi:10.1016/j.gim.2026.102537)
Supplement: Supplementary file 1 [file NIHMS2210167-supplement-All_supplemental_material.zip › 1-s2.0-S1098360026008555-mmc2.docx]

**Supplemental Material 2**

*Overall Detrimental Psychological Impact and FACToR Subscale Descriptives*

Supporting our main hypothesis, a majority of patients did not have high levels of overall DPI (n=812; 96.0%; *M*=11.04, *SD*=6.94). Most patients also had low levels of negativity (n=809; 95.6%; *M=*1.54, *SD=*2.21), uncertainty (n=795; 94.0%; *M*=2.05, *SD*=2.48), and concerns about privacy (n=806; 95.6%; *M*=0.76, *SD*=1.63). Of the high negativity patients (n=37), 83.8% had cancer (n=31), and the majority had a PLP (n=16; 51.6%). Of the high uncertainty patients (n=51), most had cancer (n=45; 88.2%), and of those, most had a VUS (n=28; 62.2%). Of those with high levels of privacy concern (n=37), most had cancer (n=34; 91.9%), and of those, most had a VUS (n=20; 58.8%). Overall, DPI, negativity, uncertainty, and privacy concern did not differ based on cancer status. Descriptively, we found support for our second hypothesis indicating that a higher frequency of patients would exhibit high uncertainty relative to the frequency of those exhibiting high overall DPI or negative emotions.

The majority of patients across all groups had high levels of positivity (n=470; 55.6%; *M*=9.07, *SD*=4.24). A chi-square analysis revealed that unaffected patients were more likely to report high positivity (high positivity: n=100; 64.1%; low positivity: n=56; 35.9%) relative to affected patients (high positivity: n=370; 53.6%; low positivity=320; 46.4%), χ^2^=5.66 (*p* = .017).

*Overall Detrimental Psychological Impact at 2-Week Baseline*

The analysis of overall DPI 2-weeks after disclosure of germline genomic testing results yielded a main effect of results type *F*(2,751)=20.90, *p*<.001, η^2^=.05, and cancer status *F*(1,751)=11.33, *p*<.001, η^2^=.02. There was not a significant interaction between the two (*p*=.444). Only ethnicity (*p*=.033) and age (*p*=.005) were significant covariates. We found that affected patients were exhibiting higher DPI than unaffected patients (*M_diff_*=2.56, *p*<.001). Additionally, within the affected patient baseline group, PLPs had higher DPI relative to VUSs (*M_diff_* =5.38, *p*<.001) and Negatives (*M_diff_* =7.01, *p*<.001), and VUSs had higher DPI relative to Negatives (*M_diff_* =1.64, *p*=.027). Within the unaffected group, PLPs also had higher DPI relative to VUSs (*M_diff_* =3.39, *p*=.018) as well as Negatives (*M_diff_* =6.06, *p*=.001), and VUSs did not differ from Negatives (*p*=.086) (See Figure 2 in Manuscript).

*Overall Detrimental Psychological Impact at 6-Month Follow-up*

While there were no main effects of results type and cancer status, nor an interaction between the two on the follow-up survey (*p*s>.105), nor any significant covariates (*p*s>.054),~~.~~ Within the affected group, PLPs once again had higher DPI than Negatives (*M_diff_* =4.55, *p*=.002), but not VUSs. Similar to baseline, VUSs also had higher DPI relative to Negatives (*M_diff_* =2.66, *p*=.008).

*Detrimental Psychological Impact Change Scores*

There were no significant differences, within subjects' main effects, or interactions on DPI across the two timepoints (*p*s > .104) after adjusting for the same covariates as in prior analyses. There was a significant between-subjects main effect of result type on DPI across timepoints, *F*(2, 368)=4.66, *p* = .010, η^2^ = .03, where PLPs (*M* = 12.45; CI = [10.52, 14.81]) exhibited significantly more DPI than VUSs (*M* = 10.11; CI = [9.22, 11.01]; *p* = .031) as well as Negative resultants (*M* = 8.08; CI = [5.98, 10.17]; *p* = .003). VUSs and Negative resultants did not differ from one another (*p* = .079). There was also a main effect of cancer status on DPI across time points, *F*(1, 368)=3.95, *p* = .048, η^2^ = .01, where affected patients had higher overall DPI across time points (*M* = 11.22; CI = [10.34, 12.10]) than their unaffected counterparts (*M* = 9.20; CI [7.41, 11.00]). Finally, significant differences were only found for affected PLPs and VUSs, whereby DPI was decreasing across time for PLPs (*M_diff_* = -1.96, *p* = .042), but increasing for VUSs (*M_diff_* =0.95, *p*=.006) (See Figure 3 in Manuscript).

*Relationship between PLP status and Sharing Results with Family*

To examine if DPI partially mediated the relationship between having a PLP vs. not and reported sharing of germline genomic testing results with family members, we conducted a mediation analysis using PLP status as the predictor variable, DPI as the mediator, and sharing of genomic test results with a family member as the outcome. Control variables included age, gender, race ethnicity, cancer status, satisfaction with care received, and understanding of provider explanation of results. In the first stage of the model, there was We found a positive association between PLP status and DPI where PLPs were more likely to be psychologically impacted relative to non-PLPs, *b* = 5.18, SE = 0.72, p < .001, CI=[3.77, 6.59]. As expected, there was also a positive association of PLP status on likelihood of sharing genomic test result information with family members, *b* = 1.06, SE = 0.27, p < .001, CI=[0.53, 1.59], indicating that PLPs were more likely to share their genomic test results with family members, relative to non-PLPs. Critically, the relative indirect effect of PLP status on likelihood of sharing genomic test results with family members, through DPI, was significant (effect = −0.30, SE = 0.08, CI [–0.47, –0.16]), indicating that having a PLP status (relative to non-PLPs) is associated with more DPI, which, in turn, was associated with a reduction in the likelihood to share test genomic test results with family members.
