# Supplementary material for: A scalable new model of germline cancer genomic care delivery: Assessing psychological outcomes
Source: Genet Med. Author manuscript; Available in PMC 2026 Oct 5. (PMC13638032; doi:10.1016/j.gim.2026.102537)
Supplement: Supplementary file 1 [file NIHMS2210167-supplement-All_supplemental_material.zip › 1-s2.0-S1098360026008555-mmc1.docx]

**Supplemental Material 1**

PMED FACToR Questions and Numbering

1) How upset did you feel about your genomic test result(s)?
3) How anxious or nervous did you feel about your genomic test result(s)?
5) How sad did you feel about your genomic test result(s)?
2) How happy did you feel about your genomic test result(s)?
4) How relieved did you feel about your genomic test result(s)?
9) How much did you feel that you understood clearly your choices for based on the genomic test result(s)?
11) How helpful was the information you received from your genomic test result(s) in planning for the future?
6) How frustrated did you feel about recommendations for your care based on the genomic test result(s)?
7) How uncertain did you feel about what your genomic test result(s) means for you?
8) How uncertain did you feel about what your genomic test result(s) means for your child(ren) and/or family’s risk of disease?
10) How concerned did you feel that your genomic test result(s) would affect your ability to get or keep health insurance?
12) How concerned did you feel that your genomic test result(s) might make it hard for you to get a job or keep a job?

Original FACToR Wording

1) How upset did you feel about your genetic test result?
2) How anxious or nervous did you feel about your genetic test result?
3) How sad did you feel about your genetic test result?
4) How happy did you feel about your genetic test result?
5) How relieved did you feel about your genetic test result?
6) How much did you feel that you understood clearly your choices for disease prevention or early detection?
7) How helpful was the information you received from your genetic test result in planning for the future?
8) How frustrated did you feel that there are no definite disease prevention guidelines for you?
9) How uncertain did you feel about what your genetic test result means for you?
10) How uncertain did you feel about what your genetic test result means for your child(ren) and/or family’s risk of disease?
11) How concerned did you feel that your genetic test result would affect your health insurance status?
12) How concerned did you feel that your genetic test result would affect your employment status?
